# Fab glycosylation of immunoglobulin G does not associate with improvement of rheumatoid arthritis during pregnancy

**DOI:** 10.1186/s13075-016-1172-1

**Published:** 2016-11-25

**Authors:** Albert Bondt, Manfred Wuhrer, T. Martijn Kuijper, Johanna M. W. Hazes, Radboud J. E. M. Dolhain

**Affiliations:** 1 Department of Rheumatology, Erasmus University Medical Center, PO Box 2400, 3000 CA Rotterdam, The Netherlands; 2 Center for Proteomics and Metabolomics, Leiden University Medical Center, 2300 RC Leiden, The Netherlands

**Keywords:** Rheumatoid arthritis, Disease activity, Inflammation, Pregnancy

## Abstract

**Background:**

Changes in immunoglobulin G (IgG) constant domain (Fc) glycosylation are associated with changes in rheumatoid arthritis (RA) disease activity in response to pregnancy. Here, we sought to determine whether the same holds true for variable domain (Fab) glycosylation.

**Methods:**

IgGs were captured from RA and control sera obtained before (RA only), during and after pregnancy, followed by Fc and Fab separation, glycan release, and mass spectrometric detection. In parallel, glycans from intact IgG were analysed. The data was used to calculate glycosylation traits, and to estimate the level of Fab glycosylation.

**Results:**

The overall level of Fab glycosylation was increased in RA patients compared to controls, while no differences in Fab glycosylation patterns were found. For the Fc and intact IgG (Total) previously observed differences in galactosylation and bisection were confirmed. Furthermore, increased galactosylation of Fc and Total were associated with lower disease activity and autoantibody positivity. In addition, the change in Fc galactosylation associated with the change in disease activity during pregnancy and after delivery, while this was not the case for Fab.

**Conclusions:**

In contrast to changes in Fc glycosylation, changes in Fab glycosylation are not associated with improvement of RA during pregnancy and arthritis flare after delivery.

**Electronic supplementary material:**

The online version of this article (doi:10.1186/s13075-016-1172-1) contains supplementary material, which is available to authorized users.

## Background

**Fig. 1:**
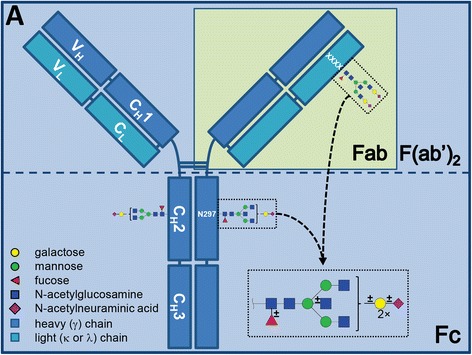
Schematic representation of immunoglobulin G. Indicated is what is generally considered as the Fc and Fab or F(ab’)_2_ portion. The glycans of both Fc and Fab consist of the same building blocks, although the most abundant glycans may differ vastly in their appearance. This figure was originally published in Bondt et al, Immunoglobulin G (IgG) Fab glycosylation analysis using a new mass spectrometric high-throughput profiling method reveals pregnancy-associated changes. *Mol Cell Proteomics*. 2014; 13:3029–3039. © the American Society for Biochemistry and Molecular Biology, and slightly adapted for the current manuscript

Rheumatoid arthritis (RA) is an autoimmune disease, for which it is well known that patients may improve during pregnancy [1]. The immunoglobulin G (IgG) fragment crystallisable (Fc) *N*-glycan compositions, specifically the levels of galactosylation and sialylation (Fig. 1), have been recognised to be different in RA patients compared to healthy controls and to be associated with RA disease activity and its improvement during pregnancy [2–4].

In addition to the Fc moiety, that always bears *N*-glycans, the hypervariable region of the antigen-binding fragment (Fab) may harbour *N*-glycans [5]. These glycans are generally present on approximately 15–25% of the Fab portions, and have been found to alter the binding properties of antibodies to their antigen, as well as effector functions, and are known to change during pregnancy [6–10]. Interestingly, for rheumatoid arthritis-specific autoantibodies (anti-citrullinated protein antibodies (ACPA)) the vast majority has been shown to carry these Fab glycans [11].

In view of the known differences between the Fc *N*-glycans of RA patients and healthy controls, and its association with disease activity and improvement during pregnancy, we aimed to determine whether similar changes and associations can be found regarding the Fab *N*-glycans. In addition, the level of Fab glycosylation was investigated.

## Methods

### Study population and data collection

For the current study we used sera from the Pregnancy-induced Amelioration of Rheumatoid Arthritis (PARA) study, a prospective cohort study on pregnancy and RA [12]. At all time points, disease activity (DAS28; based upon a count of 28 swollen and tender joints and C-reactive protein (CRP)) and medication was recorded. For the analysis we selected a subset of RA patients (n = 33) with the most pronounced (> = 0.6) or no change in disease activity, both during and after pregnancy, in search of maximum contrast. Only sera obtained before pregnancy, during the third trimester of pregnancy, and 26 weeks postpartum were analysed. In addition sera obtained from 32 healthy pregnant volunteers were analysed (third trimester and 6 months after delivery only). All patients fulfil the 1987 American College of Rheumatology criteria for RA. The study was in compliance with the Helsinki Declaration and was approved by the Ethics Review Board at the Erasmus University Medical Center, Rotterdam, The Netherlands.

### IgG capturing and *N*-glycan release

IgGs were affinity-captured in duplicate from human serum in a 96-well format, as described before, with minor modifications [9]. One set was used for IdeS digestion to separate the Fab and Fc portions, whereas the other set was kept intact to analyse the total IgG glycans. Dried samples were reconstituted in 5 μL phosphate-buffered saline and 10 μL 2% (w/v) sodium dodecyl sulfate solution, instead of 10 and 20, respectively. Similarly, PNGase F was added in 10 μL.

### Sialic acid derivatization and HILIC enrichment

To prevent the loss of sialic acids in the mass spectrometric detection of released glycans a derivatization protocol was applied as described before, with minor modifications [9, 13]. Briefly, 2 μL released glycans were added to 20 μL ethyl esterification reagent in a V-bottom 96-well plate, and incubated at 37 °C for 1 h. After the incubation 20 μL acetonitrile was added. Ethyl esterified *N*-glycans were purified from the reaction mixture using hydrophilic interaction liquid chromatography (HILIC) solid phase extraction (SPE) with a piece of cotton thread as the solid phase in a micro tip as described before [9].

### MALDI-TOF-MS and data processing

Five microliter of eluate was mixed on the spot with sodium hydroxide-spiked Super-DHB matrix (Sigma-Aldrich, St. Louis, MO, USA) on an AnchorChip plate. Automated matrix-assisted laser desorption/ionization (MALDI)-time of flight (TOF) mass spectrometry (MS) measurements were performed summing 10,000 shots per spot with 250 shot steps and a full spot random walk. Details on data processing, extraction, and the calculation of glycosylation traits are described in Additional file 1.

### Statistical analysis

Statistical analysis was performed in Stata 13SE (StataCorp, College Station, TX, USA). Bonferroni correction for multiple testing was performed throughout, with final significance thresholds depicted in the tables with results. First, a Wilcoxon rank-sum test was performed to explore the differences in glycosylation between RA patients and healthy controls outside pregnancy, at ≥26 weeks after delivery. Next we investigated which clinical covariates (use of medication, autoantibody (AAb) positivity, age at delivery and disease activity) were associated with the glycosylation in RA patients using linear regression analysis. To study changes in glycosylation over time the Wilcoxon matched-pairs signed-rank test was used, comparing pre-conception with the third trimester for the patients, and third trimester with 26 weeks postpartum for both patients and controls. Association of the changes in glycosylation over time and the accompanying changes in disease activity were explored using Spearman correlation.

## Results

### Response during pregnancy and flare after delivery

Clinical characteristics of the patients and controls are given in Additional file 1: Table S1. The patients who improved (n = 14) during pregnancy showed a mean decrease in DAS28 (delta DAS28; ΔDAS) of -1.7 (SD 0.5), whereas the nine patients that worsened showed a ΔDAS of +1.6 (SD 0.5). Ten patients did not change during pregnancy (ΔDAS = 0.0; SD 0.4). After delivery the 12 patients that flared had a mean ΔDAS of +1.5 (SD 0.8), while the 12 improving patients showed a ΔDAS of −1.5 (SD 0.6). Nine patients without a change in disease activity between the third trimester and 6 months postpartum (ΔDAS = −0.1; SD 0.4) were selected.

### Fab glycosylation is higher in RA patients outside pregnancy when compared to controls, yet with similar glycans

The calculated level of Fab glycosylation in the patients (21.4%; [IQR 19.5–25.4%]) was significantly higher than in controls (16.5%; [IQR 13.3–18.2%]) at the non-pregnant time point 26 weeks postpartum (Tables 1, 2 and 3). However, no differences between the levels of galactosylation, sialylation, fucosylation or presence of bisecting *N*-acetylglucosamine were observed for the Fab glycosylation (Tables 1, 2 and 3) compared between healthy controls and RA patients. However, for both Fc and total IgG (Total) there was a lower level of galactosylation and higher level of bisection in RA patients compared to the healthy controls.

**Table 1:** Median percentages and corresponding interquartile ranges for all time points and glycosylation traits

	Healthy				RA					
	3rd trim^a^		26 wkpp		pre-conception		3rd trim		26 wkpp	
	Median (%)	IQR	Median (%)	IQR	Median (%)	IQR	Median (%)	IQR	Median (%)	IQR
Fc										
Galactosylation	81.5	[78.1–83.2]	71.6	[67.9–74.7]	60.8	[52.2–64.3]	73.6	[66.5–79.5]	61.0	[56.8–66.7]
Sialylation	18.4	[17.1–20.5]	13.1	[11.5–15.8]	11.0	[9.0–13.1]	16.0	[14.0–19.7]	11.5	[9.4–14.1]
Fucosylation	96.8	[95.8–97.4]	97.3	[96.6–98.0]	97.9	[97.5–98.3]	97.5	[96.5–98.0]	97.7	[97.2–98.2]
Bisection	8.7	[7.3–9.7]	9.2	[8.3–10.5]	10.3	[9.0–11.5]	8.6	[7.4–10.2]	11.0	[9.5–13.4]
Total										
Galactosylation	82.9	[80.6–84.6]	74.8	[70.3–77.0]	66.0	[59.9–69.0]	77.5	[73.5–82.2]	68.6	[59.1–73.5]
Sialylation	26.2	[23.9–28.7]	21.3	[19.4–22.6]	20.8	[19.1–23.8]	27.3	[24.4–28.4]	22.6	[20.0–24.5]
Fucosylation	95.4	[94.7–96.2]	96.5	[95.5–97.2]	96.9	[96.2–97.3]	96.2	[95.4–96.7]	96.7	[96.0–97.1]
Bisection	12.6	[10.6–14.1]	14.8	[13.3–17.3]	18.1	[15.8–19.8]	15.2	[13.6–17.7]	18.1	[16.8–19.6]
Fab										
Galactosylation	96.8	[96.3–97.0]	97.2	[96.5–97.8]	96.6	[95.7–97.7]	97.3	[96.9–97.8]	96.8	[96.0–97.3]
Sialylation	81.6	[80.4–84.3]	79.0	[77.8–82.6]	79.7	[77.0–81.1]	82.7	[80.6–84.6]	80.2	[76.9–81.6]
Fucosylation	70.6	[68.0–75.3]	76.6	[74.5–80.6]	75.6	[71.9–80.5]	73.2	[61.6–75.8]	77.7	[72.8–79.7]
Bisection	37.0	[36.1–41.4]	46.6	[41.7–52.2]	51.5	[46.5–55.2]	42.1	[33.8–44.4]	52.2	[46.7–54.6]
% glycosylation	17.6	[13.7–21.0]	16.5	[13.3–18.2]	22.0	[18.7–27.4]	28.0	[23.3–32.7]	21.4	[19.5–25.4]

^a^ *Abbreviations*: *trim* trimester, *wkpp* weeks postpartum, *IQR* interquartile range

**Table 2:** Number of observations obtained for each category and time point

	Healthy		RA		
	3rd trim^a^	26 wkpp	pre-conc.	3rd trim	26 wkpp
Fc					
Galactosylation	29	25	33	31	33
Sialylation	29	25	33	31	33
Fucosylation	29	25	33	31	33
Bisection	29	25	33	31	33
Total					
Galactosylation	25	25	33	30	31
Sialylation	25	25	33	30	31
Fucosylation	25	25	33	30	31
Bisection	25	25	33	30	31
Fab					
Galactosylation	17	15	29	25	23
Sialylation	17	15	29	25	23
Fucosylation	17	15	29	25	23
Bisection	17	15	29	25	23
% glycosylation	15	15	29	24	21

^a^ *Abbreviations*: *trim* trimester, *wkpp* weeks postpartum, *pre-conc*. pre-conception

**Table 3:** *p* values obtained for comparing glycosylation traits of controls vs. cases and over time

	RA vs. healthy at 26 wkpp^a^	Healthy	RA	
		3rd trim vs. 26 wkpp	pre-conc. vs. 3rd trim	3rd trim vs. 26 wkpp
Fc				
Galactosylation	**0.000**	**0.000**	**0.000**	**0.000**
Sialylation	0.077	**0.000**	**0.000**	**0.000**
Fucosylation	0.072	**0.000**	0.003	0.048
Bisection	0.004	0.003	**0.000**	**0.000**
Total				
Galactosylation	**0.001**	**0.000**	**0.000**	**0.000**
Sialylation	0.179	**0.000**	**0.000**	**0.000**
Fucosylation	0.889	**0.000**	**0.000**	0.012
Bisection	**0.000**	**0.000**	**0.000**	**0.000**
Fab				
Galactosylation	0.347	0.333	0.004	0.148
Sialylation	0.754	0.028	**0.000**	0.007
Fucosylation	0.709	0.005	**0.000**	0.003
Bisection	0.104	0.005	**0.000**	**0.001**
% glycosylation	**0.002**	0.401	0.009	0.249

For the comparison of RA vs. healthy a significance threshold of *p* <0.0038 was used (Bonferroni corrected for 13 tests), and for the time comparisons *p* <0.0013 (corrected for 42 tests). Significant observations are highlighted in bold font

^a^ *Abbreviations*: *wkpp* weeks postpartum, *trim* trimester

### Galactosylation of Fc and intact IgG, but not Fab, associate with disease activity and autoantibody positivity

In addition, it was explored which clinical covariates were associated with the levels of the calculated glycosylation traits at 26 weeks postpartum. Covariates were first tested univariate, and those with *p* <0.2 were included in the multivariate analysis. No Fab glycosylation models remained significant after Bonferroni correction. However, for galactosylation of both Fc and Total multivariate models could be composed with R^2^ values of 0.4 and 0.5, respectively. For these models the main contributing factors were disease activity (beta = −0.5) and autoantibody positivity (beta = +0.4). The results for all multivariate models are shown in Additional file 1: Table S2.

### Fab glycosylation changes during pregnancy and after delivery are not associated with changes in disease activity

**Fig. 2:**
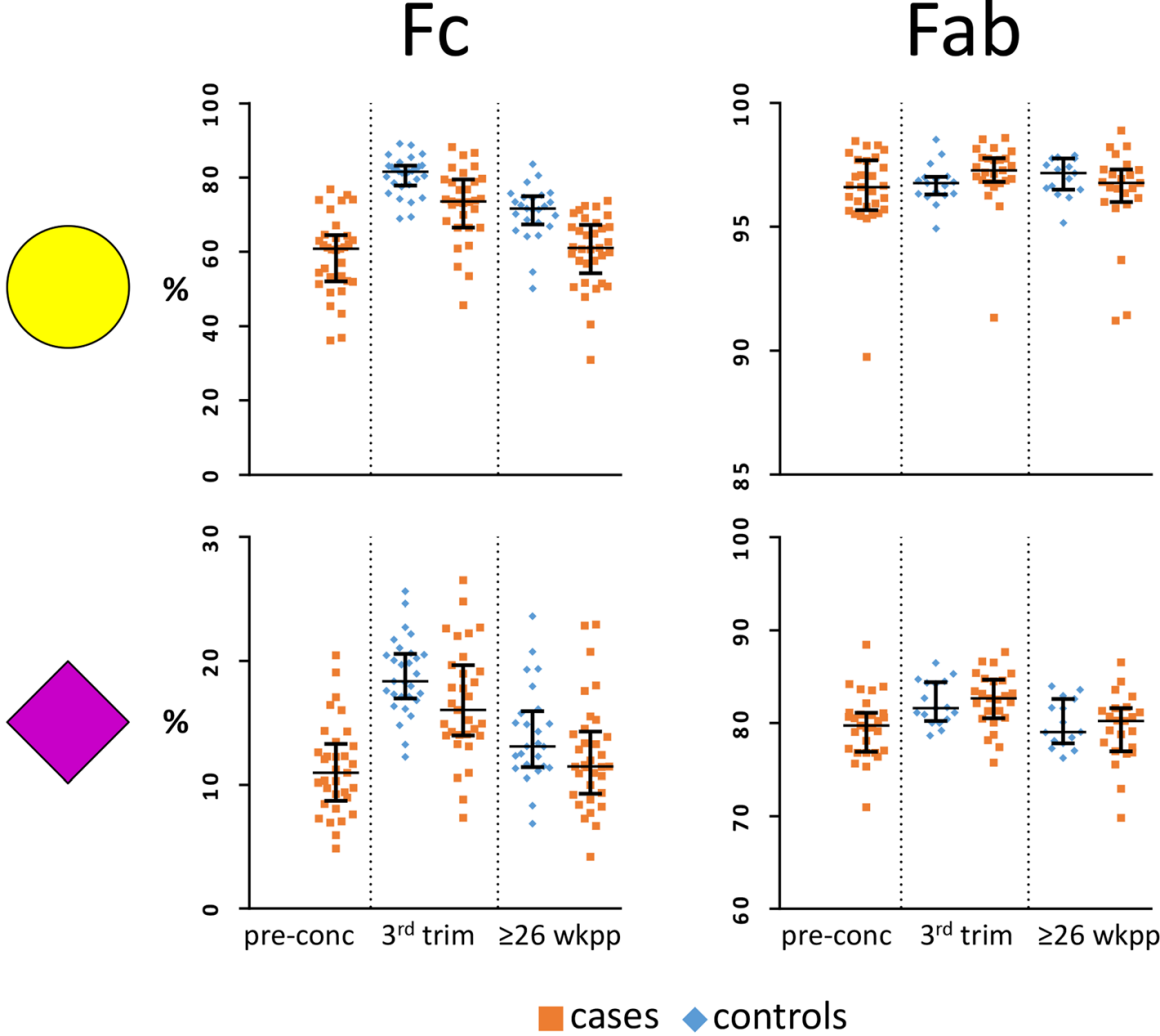
Fc and Fab galactosylation and sialylation in RA and healthy controls. The percentages of galactosylation (*yellow circle*) and sialylation (*purple diamond*) on the **Fc** show a clear increase during pregnancy (from pre-conception to the 3rd trimester; RA only) and decrease after delivery (from 3rd trim to 26 weeks postpartum), as well as differences between cases and controls. No differences between cases and controls, and only minor pregnancy-associated changes were observed for the **Fab** glycosylation. *Abbreviations*: *pre-conc* pre-conception, *trim* trimester, *wkpp* weeks postpartum

Galactosylation and sialylation of Fab, Fc and Total increased between pre-conception and the third trimester of pregnancy in RA patients (Tables 1, 2 and 3; Fig. 2). The opposite was observed after delivery in both patients and healthy controls, except for the Fab portion where only a decrease in sialylation was observed. For fucosylation and bisection a decrease during pregnancy (RA only) and increase after delivery was observed for Fab, Fc and Total, in both patients and controls (Tables 1, 2 and 3). In addition, during pregnancy an increase in the level of Fab glycosylation was found in RA patients. Of note, changes in glycosylation traits that were high, e.g. Fab galactosylation and Fc fucosylation, were often not significant after Bonferroni correction (Tables 1, 2 and 3; Fig. 2).

The Fc galactosylation change from pre-conception to the third trimester of pregnancy was negatively associated with the change in disease activity in that time span (rho = −0.39, *p* = 0.029; Additional file 1: Table S3, Additional file 1: Figure S1). The opposite was observed for the change Fc and Total galactosylation from third trimester to 26 weeks postpartum (rho = 0.48 and 0.62, *p* = 0.006 and 0.001, respectively). For neither the Fab glycosylation traits nor the Fab glycosylation level these associations were observed.

## Discussion

There is a strong association of IgG Fc galactosylation with RA disease activity and the pregnancy-associated improvement thereof [2, 3]. For the Fab portion of IgG this has not yet been investigated. Therefore, we applied our recently developed method for the analysis of Fab glycosylation on samples from a set of RA and healthy individuals in the context of pregnancy. Interestingly, we found no qualitative differences in Fab glycosylation between cases and controls nor an association with changes in disease activity when we compared several glycosylation traits. This may in part be caused by the fact that Fab galactosylation and sialylation levels are already high in the non-pregnant state, leaving less room for a potential pregnancy-associated increase.

Since the Fab portion of IgG – in contrast to the Fc portion – is not always glycosylated, the association of changes in Fab glycosylation with disease activity could be quantitative rather than qualitative. In line with this, it has recently been published that the RA-specific ACPA autoantibodies express high levels of Fab glycosylation [11]. Indeed, in this study we did observe increased levels of Fab glycosylation. However, even though the levels are higher in RA patients compared to controls, they were not associated with disease activity.

In the current study we chose to include only a limited number of patients and time points. Therefore minor associations between changes in RA disease activity and changes in Fab glycosylation could have been missed.

## Conclusions

In conclusion, this study clearly demonstrates that, in contrast to changes in the glycosylation of the IgG Fc domain, changes in the glycosylation of the IgG Fab domain do not seem to play a major role in the pregnancy-associated improvement of RA.

## Additional files

Additional file 1: Additional Methods, Figure and Tables. The additional file contains an addition to the Methods, a figure, and three additional tables. (DOCX 262 kb)
Additional file 2: Data used for analysis. Contains the relative abundances of the observed glycans and the calculated glycosylation traits for the RA patients and healthy controls used in the current study. (XLSX 120 kb)

## Abbreviations

AAb: autoantibody
ACPA: anti-citrullinated protein antibodies
DAS28: based upon a count of 28 swollen and tender joints
Fab: antigen-binding fragment
Fc: fragment crystallisable
HILIC: hydrophilic interaction liquid chromatography
IgG: immunoglobulin G
MALDI: matrix-assisted laser desorption/ionization
MS: mass spectrometry
PARA: Pregnancy-induced Amelioration of Rheumatoid Arthritis
RA: rheumatoid arthritis
SPE: solid phase extraction
TOF: time of flight
