# Supplementary material for: Fab glycosylation of immunoglobulin G does not associate with improvement of rheumatoid arthritis during pregnancy
Source: Arthritis Res Ther. 2016 Nov 25;18:274. doi: 10.1186/s13075-016-1172-1 (PMC5123206; doi:10.1186/s13075-016-1172-1)
Supplement: Additional file 1: — Additional Methods, Figure and Tables. The additional file contains an addition to the Methods, a figure, and three additional tables. (DOCX 262 kb) [file 13075_2016_1172_MOESM1_ESM.docx]

Additional file 1 – Additional Methods, Additional Figure 1 and Additional Tables 1-3

*Addition to ‘Fab glycosylation of immunoglobulin G does not associate with improvement of rheumatoid arthritis during pregnancy’ by Albert Bondt et al.*

Methods

Bruker data files were exported to .XY files using MassyTools version 0.1.8.0.^1^ Calibration was performed using the following list of calibrants, depicting the composition of the glycan and the theoretical *m/z* value of the highest isotopic peak:

H3N4F1 1485.5337

H4N4F1 1647.5865

H5N4F1 1809.6393

H5N4E1 1982.7081

H5N4F1E1 2128.7660

H5N4E2 2301.8348

H5N5F1E1 2332.8487

H5N4F1E2 2448.8960

H5N5F1E2 2651.9754

MassyTools was set to only use glycans with a signal-to-noise ratio (S/N) >9 for calibration, and to use at least 5 calibrants from the list. Twenty-five glycan compositions which were identified were extracted from the spectra for further use.

Data processing and calculation of glycosylation traits

Glycans that were present in at least 40% of the spectra of either RA patient or healthy IgG, Fc or Fab (six subgroups) were included in the final calculations of relative abundances and the glycosylation traits. The presence of a glycan was defined as S/N>9, ppm error <10, and deviation from the theoretical isotopic pattern < 1%. Furthermore, spectra with a total intensity of less than 2500 and a ‘Fraction of Analyte Area – Background Area above S/N cut-off (9)’ lower than 87% were excluded.

Finally, from the relative abundances the percentage of galactosylation, sialylation, bisection and fucosylation were calculated. Galactosylation = (0.5 × sum(H4*)) +sum( H5*). Sialylation = (0.5 × sum(*E1)) + sum(*E2). Bisection = sum(*N5*). Fucosylation = sum(*F1*).

In addition, the level of Fab glycosylation was calculated. The relative abundance of Fab specific glycans in the Total IgG spectra was divided by the relative abundance of the same glycans in the Fab spectra. This ratio was divided by the ratio of Fc specific glycans in the Total spectra compared to the Fc specific glycans in the Fc spectra.

((((H5N5F1E2IgG+H5N5F1E1IgG+H5N4F1E2IgG) / (H5N5F1E2Fab+H5N5F1E1Fab+H5N4F1E2Fab)) / ((H3N4F1IgG+H4N4F1IgG+H5N4F1IgG) / (H3N4F1Fc+H4N4F1Fc+H5N4F1Fc)))*100

A glycan was considered Fab specific if the relative abundance in Fab spectra was at least 20× higher compared to Fc spectra, and relative abundance in Total spectra at least 3× higher than in Fc spectra. Using similar rational the Fc specific glycans were established.

1. Jansen BC, Reiding KR, Bondt A, et al. MassyTools: A high throughput targeted data processing tool for relative quantitation and quality control developed for glycomic and glycoproteomic MALDI-MS. J Proteome Res 2015;**14**(12):5088-98.

Additional Figure 1


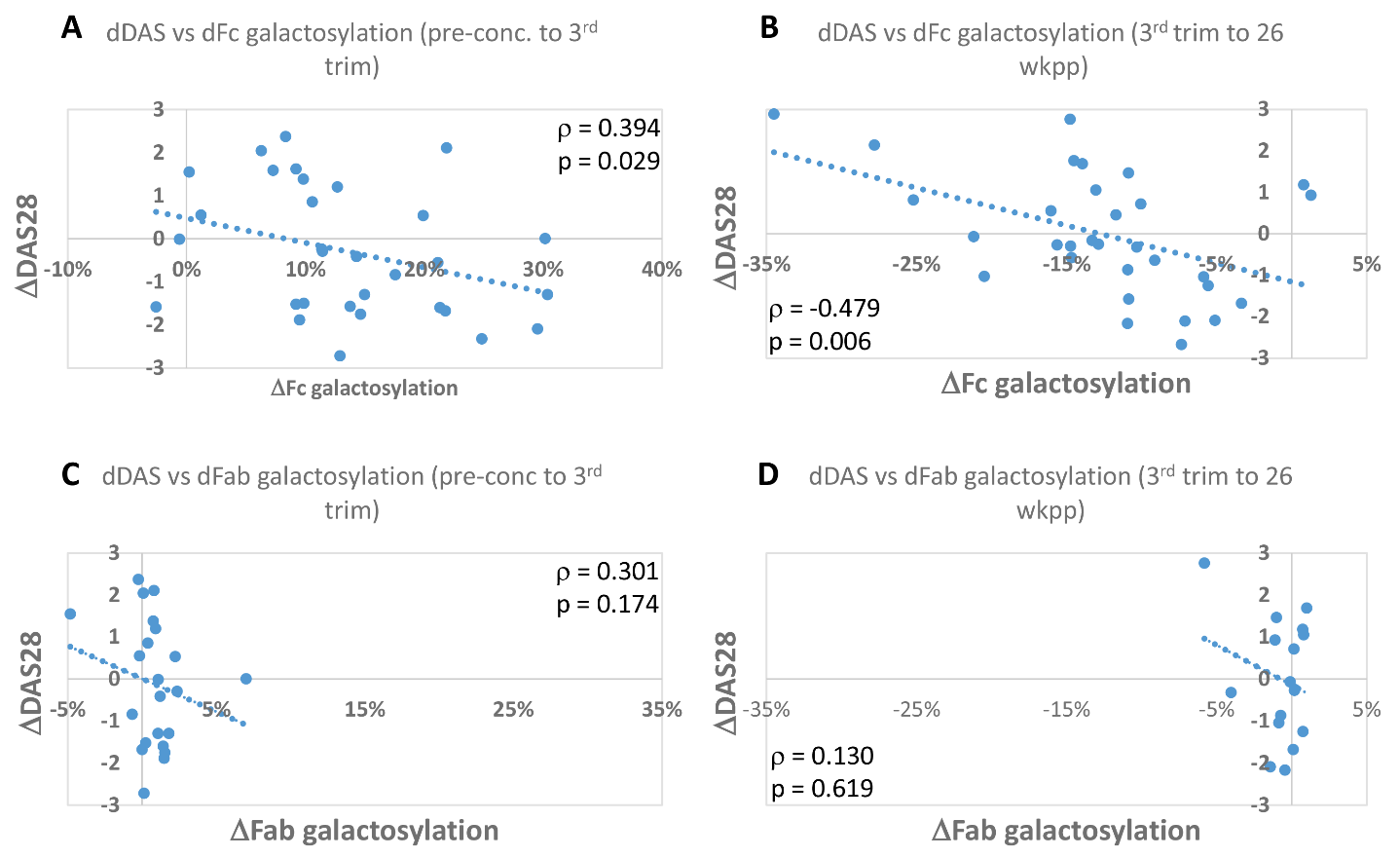


**Additional Figure 1 Association of ΔDAS28 and ΔGalactosylation.** Changes in disease activity (ΔDAS28) during pregnancy (**A**, **C**) and after delivery (**B**, **D**) associate with changes in Fc but not Fab galactosylation. Abbreviations used: pre-conc. = pre-conception; trim = trimester; wkpp = weeks postpartum.

**Additional Table 1 Cohort characteristics**

|  | **Controls** (n=29) | **Patients** (n=33) |  |  |
| --- | --- | --- | --- | --- |
| Mean age at delivery in years (SD) | 32.1 (4.1) | 32.6 (4.0) |  |  |
| Mean disease duration in years at  first visit (range) |  | 6.28 (0.27-24.73) |  |  |
| Serological status |  |  |  |  |
| *ACPA positive, n (%)* |  | 22 (67) |  |  |
| *RF positive, n (%)* |  | 24 (73) |  |  |
| Erosive disease, n (%) |  | 26 (81) |  |  |
| Response during pregnancy |  |  |  |  |
| *good/moderate response, n (%)* |  | 14 (42) |  |  |
| *no response, n (%)* |  | 10 (30) |  |  |
| *deterioration, n (%)* |  | 9 (27) |  |  |
| Flare after delivery |  |  |  |  |
| *severe/moderate deterioration, n (%)* |  | 12 (36) |  |  |
| *no deterioration, n (%)* |  | 9 (27) |  |  |
| *amelioration, n (%)* |  | 12 (36) |  |  |
|  |  | *pre-conc* | *3^rd^ trim* | *26 wk pp* |
| Mean DAS28 at each time point (SD) |  | 3.85 (1.0) | 3.56 (1.3) | 3.52 (1.4) |
| Medication at each time point, n (%) |  |  |  |  |
| *metotrexate* |  | 0 | 0 | 10 (30) |
| *sulfasalazine* |  | 12 (36) | 10 (30) | 8 (24) |
| *hcq* |  | 2 (6) | 0 | 3 (9) |
| *prednisone* |  | 10 (30) | 15 (45) | 13 (39) |
| *biologicals* |  | 0 | 0 | 5 (15) |

**Additional Table 2**

**Multivariate exploration of clinical covariates associating with IgG glycosylation**. Models that remain significant after Bonferroni correction (p <0.05/13) are indicated by an asterisk (*).

|  |  | **Model details** | | | Covariates included^1^ | | | | | | | | | | | | | | | |
| --- | --- | --- | --- | --- | --- | --- | --- | --- | --- | --- | --- | --- | --- | --- | --- | --- | --- | --- | --- | --- |
|  |  |  |  |  | Use of prednisone | | Use of methotrexate | | Use of sulfasalazine | | Use of hydroxychloroquine | | Use of biologicals | | Autoantibody positivity | | Age at delivery | | DAS28CRP | |
|  |  | n | R^2^ | p | p | beta | p | beta | p | beta | p | beta | p | beta | p | beta | p | beta | p | beta |
| **Fc** | Galactosylation | 33 | 0.415 | 0.004* | 0.256 | -0.183 | 0.553 | -0.091 |  |  |  |  |  |  | 0.026 | 0.353 |  |  | 0.002 | -0.512 |
|  | Sialylation | 33 | 0.101 | 0.072 |  |  |  |  |  |  | 0.072 | 0.317 |  |  |  |  |  |  |  |  |
|  | Fucosylation | 33 | 0.164 | 0.068 |  |  |  |  |  |  | 0.447 | -0.135 |  |  |  |  | 0.059 | -0.343 |  |  |
|  | Bisection | 33 | 0.183 | 0.049 | 0.071 | -0.314 |  |  |  |  | 0.045 | 0.351 |  |  |  |  |  |  |  |  |
| **Total** | Galactosylation | 31 | 0.483 | 0.000* |  |  | 0.159 | -0.204 |  |  |  |  |  |  | 0.004 | 0.439 |  |  | 0.001 | -0.525 |
|  | Sialylation | 31 | 0.273 | 0.012 |  |  |  |  |  |  |  |  |  |  | 0.009 | 0.461 | 0.247 | 0.192 |  |  |
|  | Fucosylation | 31 | 0.169 | 0.075 |  |  |  |  |  |  |  |  |  |  | 0.156 | -0.253 | 0.104 | -0.292 |  |  |
|  | Bisection | 31 | 0.227 | 0.027 |  |  |  |  |  |  |  |  |  |  | 0.023 | 0.403 | 0.231 | 0.205 |  |  |
| **Fab** | Galactosylation | 23 | 0.354 | 0.082 | 0.318 | -0.235 | 0.304 | -0.218 | 0.218 | 0.247 |  |  |  |  |  |  |  |  | 0.330 | -0.225 |
|  | Sialylation | 23 | 0.205 | 0.215 | 0.340 | -0.241 | 0.341 | -0.217 |  |  |  |  |  |  |  |  |  |  | 0.605 | -0.125 |
|  | Fucosylation | 23 | 0.315 | 0.061 |  |  |  |  |  |  | 0.248 | -0.250 | 0.057 | 0.391 |  |  | 0.473 | -0.153 |  |  |
|  | Bisection | 23 | 0.416 | 0.015 |  |  |  |  |  |  | 0.029 | -0.428 |  |  | 0.033 | 0.416 |  |  | 0.045 | 0.376 |
|  | % glycosylation | 21 | 0.419 | 0.024 |  |  |  |  | 0.162 | -0.271 |  |  |  |  | 0.125 | 0.305 | 0.034 | 0.434 |  |  |

^1^ Covariates were first tested univariate. Covariates with a univariate p<0.2 were included in the multivariate analysis per glycosylation trait.

**Additional Table 3**

Differences in DAS28 and glycosylation traits were calculated between the pre-conception time point and 3^rd^ trimester (during pregnancy), and between 3^rd^ trimester and 26 weeks postpartum (after delivery). A Spearman’s rank correlation test was performed to study the association between de change in DAS28 and the changes in glycosylation. P-values < 0.05 are depicted in bold font, and significance after Bonferroni is indicated by an asterisk.

|  | Association of delta DAS28 with delta ...: | during pregnancy | | after delivery | |
| --- | --- | --- | --- | --- | --- |
|  |  | rho | p | rho | p |
| **Fc** | Galactosylation | -0.394 | **0.029** | -0.479 | **0.006** |
|  | Sialylation | -0.319 | 0.081 | -0.125 | 0.504 |
|  | Fucosylation | 0.153 | 0.412 | -0.067 | 0.721 |
|  | Bisection | -0.113 | 0.547 | -0.075 | 0.690 |
| **Total** | Galactosylation | -0.263 | 0.161 | -0.617 | **0.001^*^** |
|  | Sialylation | -0.119 | 0.533 | -0.100 | 0.612 |
|  | Fucosylation | -0.024 | 0.901 | -0.127 | 0.520 |
|  | Bisection | 0.087 | 0.649 | 0.120 | 0.544 |
| **Fab** | Galactosylation | -0.301 | 0.174 | 0.003 | 0.991 |
|  | Sialylation | -0.333 | 0.131 | 0.032 | 0.905 |
|  | Fucosylation | 0.140 | 0.536 | -0.056 | 0.837 |
|  | Bisection | 0.158 | 0.484 | -0.015 | 0.957 |
|  | % glycosylation | 0.195 | 0.397 | 0.121 | 0.694 |
